# Roles, Facilitators and Challenges of Employment Support Specialists Assisting Young People with Mental Health Conditions

**DOI:** 10.1007/s10926-020-09930-x

**Published:** 2020-10-22

**Authors:** Janhavi Ajit Vaingankar, Wen Lin Teh, Kumarasan Roystonn, Janrius Goh, Yun Jue Zhang, Pratika Satghare, Shazana Shahwan, Siow Ann Chong, Swapna Verma, Zhuan Liang Tan, Benjamin Tay, Yogeswary Maniam, Mythily Subramaniam

**Affiliations:** 1 Research Division, Institute of Mental Health, 10 Buangkok View, Singapore, 539747 Singapore; 2 Department of Psychosis, Institute of Mental Health, 10 Buangkok View, Singapore, 539747 Singapore; 3 Sector Strategy Group, National Council of Social Service, 170 Ghim Moh Road #01-02, Singapore, 279621 Singapore

**Keywords:** Mental disorders, Employment, Qualitative research, Vocational guidance, Asia

## Abstract

*Purpose* The present study aimed to understand the roles, effective strategies and facilitators, and challenges of employment support specialists (ESS) in assisting young people with mental health conditions (MHCs) gain and sustain employment in Singapore. *Methods* An interpretative qualitative design using an inductive approach was adopted for this study. Using a semi-structured interview guide, in-depth interviews were conducted with twenty ESS employed with mental health service providers or other community-based centers. Verbatim transcripts of the interviews were thematically analyzed using inductive methods. ESS were broadly classified as “any professionals providing employment-related support to people with MHCs”. *Results* Majority of the ESS were employed at a tertiary psychiatric institute. Participants included vocational and occupational specialists, case managers and other clinical professionals. Three key themes emerged from the data: (i) descriptions of roles undertaken by the ESS depicting a wide range of services and requisite skillsets; (ii) facilitators that benefit young people with MHCs’ in terms of job placement, for example, ESS’ attitudes and attributes, and their clients’ disposition; and (iii) challenges that deter effective job placements, such as factors pertaining to the ESS themselves, their clients, and clients’ employers. Under this theme, ESS also proposed ways to improve employment opportunities of people with MHCs. *Conclusions* This study provided insight into a range of tasks performed and challenges faced by ESS in Singapore while assisting their clients. There is a need to address ESS’ challenges and expectations in order to enhance their efficiency and aid reintegration of young people with MHCs into the workforce and the society.

**Electronic supplementary material:**

The online version of this article (10.1007/s10926-020-09930-x) contains supplementary material, which is available to authorized users.

## Introduction

Employment of people with mental health conditions (MHCs) remains a challenge across the world [1], and significantly so in conservative socio-cultural settings where considerable stigma associated with MHCs still prevails [2, 3]. Studies have shown that young adults face an uphill task while entering the labor market and are a particularly vulnerable group due to their lack of adequate training and skills requisite for competitive job placements [4]. This difficulty is further aggravated if they have MHCs, which research has consistently linked with increased risks of marginalization faced by this group of young adults [4]. On the other hand, studies assessing the impact of employment on people with MHCs have uncovered several positive outcomes and experiences. It is proposed that employment not only helps young adults initiate internal change, such as developing self-confidence, and gaining deeper understanding of the situations they are in, but also empowers their personal recovery process [4]. In the long term, employment is also believed to be associated with improvements in social and occupational functioning, self-esteem and self-management of mental conditions, and reduction in psychiatric symptoms, and healthcare utilization of people with MHCs [5–9].

It is now widely accepted that promoting employment for people with MHCs entails far-reaching benefits to them and society as a whole. Measures to promote employment for this population generally aim to minimize the impact of barriers such as stigma and improve social inclusion via community-based interventions, services and legislation [10]. Employment-focused services such as traditional vocational rehabilitation and supported employment are routinely applied to improve the employability of people with MHCs through imparting job readiness skills and training in terms of their capacity, skills and outreach. While the definitions and models of such employment support vary in terms of clientele, resources and regions, the overall goal is to allow relatively rapid job placements and longer job retention using strategies customized to clients’ needs and strengths [11–14]. A more recent model of employment support for people with MHCs, the individual placement and support (IPS) supported employment programme recommends that ESS are fully integrated within community mental health services comprising multiple professionals ranging from clinical treatment teams to employment specialists and vocational rehabilitation counselors [15, 16]. People with MHCs placed via such models have been consistently shown to achieve superior employment outcomes compared to their counterparts receiving standard clinical care alone [11, 17–20]. As a result, employment support services are increasingly considered a critical component within the mental healthcare system [21].

While the evidence on effectiveness of employment support interventions for PMHCs is growing and is largely positive, most of this work has been conducted in Western populations. Very few studies have delved into employment support relevant to people with MHCs in Asia [22] and even fewer, if any, have explored experiences and perspectives of employment support specialists (ESS) assisting people with MHCs with employment.

Singapore is a developed economy in South-East Asia, with a predominantly multi-ethnic Asian population of 5.6 million, and a very low unemployment rate of 2.2% [23]. In the recent years, a range of community-wide initiatives were launched to improve employment rates among vulnerable populations, including people with MHCs and to reduce the associated stigma. To enable occupational inclusion, the Ministry of Manpower of Singapore promotes the Tripartite Alliance for Fair and Progressive Employment Practices (TAFEP) guidelines that provide recommendations on wages, fair employment practices and hiring people with medical conditions including MHCs [24]. Recognizing the role of employment in fostering greater independence and social inclusion for people with MHCs, the National Council of Social Service has also initiated ‘Project H.I.R.E.’ (Help Integrate Recovering persons with mental health issues through Employment) geared at increasing employment opportunities for people with MHCs. This initiative aims to establish partnership and co-create solutions with multi-sectoral stakeholders such as community partners, social service agencies, private and national agencies and other social service agencies to enhance awareness on employability of people with MHCs, and enhance job readiness and workplace inclusiveness support for them [25]. Moreover, the emphasis on mental health issues is expected to grow. For example, the President’s Challenge, an annual community outreach and fund-raising campaign, will centre on helping those with mental conditions next year while continuing its support for a broad range of social causes. In her address, the President declared $10M funding to social service agencies assisting people with MHCs return to the workplace and appealed to employers to be more open to providing equal work opportunities to this group [26, 27]. Given these initiatives, gathering opinions from ESS—among other stakeholders—within the mental health sector and in the local context of Singapore will be critical for devising new strategies and processes to aid people with MHCs’ pathway to employment.

At a national tertiary psychiatric institution in Singapore, a ‘Hybrid Supported Employment Program’ based on principles of IPS is adopted. This model focuses on vocational rehabilitation and reintegration of people with psychiatric problems. The team comprises occupational therapists, medical social workers, case managers with psychology background and job placement officers, broadly termed as vocational specialists who perform work readiness assessments, workplace modifications, employer education and ongoing jobsite support for clients [28]. The program initially covers transitional employment skills training in areas such as administrative support, sales or other service industries, with the aim to empower and facilitate gradual return to work through supported employment. Upon completion of the requisite training phase, clients are transferred to the supported employment programme whereby assistance is provided for job search, placements and other support. In this process, close communication is maintained with clients’ clinical teams such as case managers and psychiatrists. The supported employment team comprises vocational specialists and job placement officers who maintain a database of employers from the community who offer work opportunities. Clients have a choice to avail themselves to the options available in the database, or seek assistance in other aspects such as interview preparedness or vocational guidance while they seek jobs by themselves. Besides the ESS teams based in mental health settings, there are various community service centres and voluntary welfare organizations that support job placements for people with mental and physical disabilities using their own processes and models of employment support. In addition to the specialized vocational rehabilitation teams, other mental health professionals such as case managers, medical social workers and clinicians (psychiatrists) are often involved in advice, assessment or support for employment of their clients.

To the best of our knowledge, in Singapore, research in the area of employment of people with MHCs is limited, with existing studies focusing mainly on public attitudes towards stigma and employment of people with MHCs [2, 3]. Therefore, there is a sizable knowledge gap in implementing and enhancing support services that cater to a wider range of service providers. This study aimed to first gain a deeper understanding of ESS’ roles in assisting young people with MHCs with employment, and then explore strategies, facilitators and challenges from their perspective. The term ESS indicates a broad group professionals covering trained vocational or occupational therapists and other service providers such as case managers, counselors, social workers and clinicians.

## Methods

### Ethical Aspects

This study was approved by the Domain Specific Review Board of the National Healthcare Group. Written informed consent was obtained from all participants prior to the interviews which included consent to audio-record the interviews.

### Study Design

An exploratory qualitative study using an interpretative epistemological approach was conducted in 2017. This was the first study to explore ESS experiences in Singapore as there were no pre-exiting theories around roles and challenges faced by ESS in an Asian setting. Hence, an interpretative study design allowed in-depth understanding from the ESS’ perspectives. The current study was part of a larger mixed methods study that aimed to obtain multi-stakeholder perspectives on employment of young people with MHCs. For the purpose of this study, MHCs referred to diagnosed psychiatric conditions that included a broad spectrum of conditions such as psychoses or schizophrenia, mood, anxiety disorders, or disorders such as addictions, and excluded developmental disorders, intellectual disabilities or severe mental and neurological disabilities. Qualitative data collected from interviews with the ESS were used in the present study.

### Sampling

Purposive sampling using a maximum variability approach was adopted to obtain views from a wide range of ESS working at diverse organizations including psychiatric hospitals, social service agencies, community social service centers, and institutes of higher learning. To ensure information-rich data, sampling sought heterogeneous participant groups based on their place of work, years of experience, age and gender. Employment related support provided by the ESS included any tasks such as vocational training, job matching, placement, skills training or employer relations, and mitigation. Self-referrals for participants were initially sought through a mass email from staff at a tertiary psychiatry institute, following which snowball sampling was used to engage ESS working in other organizations. Study participants were requested to inform their peers about the study. To facilitate this process, they were provided with a brochure that included study information and contact details of study team members. Interested ESS directly approached the team members, who checked whether they fit the purposive criteria and eligibility. Sampling was discontinued after reaching data saturation.

### Participants

The ESS were broadly defined as any persons providing employment-related support to young people with MHCs, who were in their late teens to early adulthood, roughly in the ages of 18–35 years. This age group was chosen considering a target population with at least higher secondary level education and reflecting clients in the “youth” population as defined by Singapore’s National Youth Council. For the purpose of this study, experiences of ESS were in relation to clients with MHCs such as schizophrenia, mood and anxiety spectrum disorders while developmental disorders or learning disabilities were excluded. ESS were included in the study if they were 21 years of age and above and involved in any of the above tasks on a regular basis. A total of 20 ESS were interviewed. ESS included professionals employed with healthcare institutions: seven vocational or occupational specialist who were occupational therapists and involved in assessment for job suitability, long term vocational coaching and job placements and eight clinical professionals such as case managers and medical social workers working in mental health settings who held other primary job roles but performed several tasks relating to job placements for their clients. Five of the participants were employed in non-healthcare services, such as voluntary welfare organizations and academic institutes.

### Interview Procedure

A semi-structured interview guide (Supplementary File 1), was designed to address key aspects around the employment of people with MHCs. Narratives of participants were elicited via probes. Appropriate prompts were incorporated to provide flexibility and allow other topics to emerge inductively. Face-to-face interviews were conducted by six of the authors who are trained researchers and experienced in qualitative methodology. The interviewers first asked general introductory questions to obtain participants’ personal background, such as education and other training, and occupational backgrounds, and encourage participant engagement, before moving on to ESS’ experiences. Participants were given adequate time to share their experiences. Interviews lasted from 26 to 90 minutes and were audio-recorded. All interviews were conducted in English and transcribed verbatim. Interview content was reviewed concurrently with data collection to assess data saturation, which was the point where no new content came up in the interviews.

### Coding and Data Analysis

This study followed an interpretative approach where interviews were used to understand how participants perceived their experiences and how they chose to report them. An inductive approach [29] was undertaken by four of the study authors who also performed the coding and analysis. NVivo 11 [30] was used to code and organize the data. The coders familiarized themselves with the content, contributed to the development of the interview guide, transcribed and/or verified the content of their own interviews, and by reading through all transcripts. A few transcripts were first read through by the coders to perform open coding and generate a preliminary coding framework. These were later combined based on group consensus to develop a codebook. As coding was team-based, simplified definitions were developed for the codes using group consensus prior to coding of the data [31]. The coded content was re-visited upon completion of coding and read multiple times to identify salient themes based on primacy (content volunteered by the interviewee first after each question), intensity (the content that interviewee speaks about for a relatively long period of time), and frequency (the content that is repeated across interviews) [32]. These themes were combined based on their interrelation and co-occurrence in the interviews to produce themes of relevance to the ESS in relation to providing support to young people with MHCs. Throughout the analysis, authors identified themes that fit the majority. Any discrepancies in the interpretation of the definitions or pattern of coding were checked by independently coding one transcript by all coders and deliberating on the variations until a consensus was reached.

## Results

The average age of the ESS was 43 years old, with the youngest being 27 and oldest 67, and work experience ranging from 3 to 38 years in this field. Majority had tertiary educational qualifications, while two held vocational diploma certificates. Table 1 provides details on participants’ background.

**Table 1:** Characteristics of employment support specialists working with young people with MHCs (n = 20)

	N (%)
Gender	
Men	8 (40%)
Women	12 (60%)
Education	
Vocational diploma	2 (10%)
University and above	18 ( 90%)
Occupation	
Vocational/occupational specialist	7 (35%)
Other employment support specialist	5 (25%)
Clinical team member (Counsellor/Social worker/Clinician/Case manager)	8 (40%)
Formal training in providing employment services to people with MHCs	15 (75%)

Themes and subthemes that emerged from the interviews with the ESS are listed in Table 2. Three key themes that emerged from the interviews were:

**Table 2:** Themes related to employment of young people with MHCs

Theme	Sub theme	Subcategory
Role description of the ESS	(i) Vocational	a. Profiling b. Coaching and counselling c. Job preparation and placement d. On-the-job support e. Employer relations
	(ii) Non-vocational	a. Client management b. Engage community partners
Facilitators of young people with MHCs’ employment	(i) ESS-related	a. Stewardship b. Client-centeredness
	(ii) Client-related	a. Disposition and traits b. Illness perception c. Support network
	(iii) Other	
Challenges in young people with MHCs’ employment	(i) ESS-specific	a. Balancing expectations b. Identity struggle c. Dilemma of disclosure
	(ii) Client-specific	a. Managing job expectations b. Sustaining work
	(iii) Employer-specific	a. Faced by young people with MHCs and ESS b. Faced by employers

*MHCs: Mental health conditions; ESS: Employment Support Specialists*

1. Descriptions of roles undertaken by the ESS; narratives depicted a range of vocational and non-vocational tasks.
2. ESS’ perspectives on facilitators and skill sets that aid people with MHCs’ job placement. These included attributes of the ESS such as stewardship and client centeredness that were expressed as being helpful in securing job placements and service provision, client-related factors such as illness perception, traits and attitudes towards employment and other facilitators such as governmental and non-governmental initiatives in the community and technological advances such as the use of social media platforms that allowed youth to stay connected and thereby increase their access to employment resources.
3. Experiences shared in relation to the challenges and limitations faced by the ESS that hampered effective service delivery, including aspects pertaining to the ESS themselves, their clients, and employers. Under this theme, ESS also spoke about the dilemma they faced in relation to disclosing their clients’ mental condition to employers and proposed ways to improve employment and career opportunities for the people with MHCs.

### Theme 1: Role Description

Given the multidisciplinary composition of the study sample that included vocational or occupational therapists and other professionals engaged, a wide array of roles was mentioned by the ESS. Broadly, ESS’ performed tasks directly related or limited to their clients’ employment, termed as ‘vocational’ roles and other tasks.

The ESS described how they directly mediated the employment journey of the people with MHCs. These covered equipping clients with transitional employment skills and assisting them with job search, placements and other support. As expected, these tasks formed the bulk of their workload and covered a range of services starting with their first contact with the clients and to their discharge from services. The duration and frequency of service provision varied widely; while some ESS had predefined criteria for discharge from services, others mentioned the decision was subjective and often prolonged. Based on the narratives of the ESS, ‘**profiling**’ was an essential first step, whereby the ESS engaged in discussions with the people with MHCs to understand their backgrounds, job preferences, motivations and conduct capacity assessments in relation to their symptoms, treatments and skills. Describing this process, ESS mentioned,

“I need to know quite a fair bit in terms of their background; like their educational background and their employment history also.” (ESS20/F).

“It can be physical assessment. Like stamina, how their endurance level is like. It can be cognitive assessment. How well they take instructions. Their memory. Or it can be erm, even how they move about in a community. How they take bus, how they take public transport and all these (things). And sometimes we will invite their caregivers, family members to actually join in also. To actually understand how they function at home.” (ESS02/F).

As part of enhancing clients’ ‘**job-preparation and transitional employment skills**’, ESS performed a range of tasks which included training on interview skills through role play, assisting with job search and resumes, or working on clients’ physical fitness. For example, for jobs involving standing for long hours such as in the service industry, the ESS enlisting clients for strength building exercises. If necessary, and upon consultation with the clients, some also provided psychoeducation to their potential employers and future colleagues, and when needed, ESS also participated in discussions around fair remuneration and negotiations on the job scope. In essence, ESS worked towards preparing their clients in the daily operations of possible employments and aiding them to be independent workers.

“They can choose to come for our employment training class whereby it is a six weeks’ program. Just half a day, going through with them a bit about their illness management, recovery, work skills like in terms of preparing them for interviews, writing CVs, and some workplace communication skills as well.” (ESS17/F).

Following this stage, ESS get engaged in provision of supported employment. ESS narrated how their role in **placement and on-the-job support** whereby ESS closely followed their client’s progress in their workplaces, helped de-escalate stressful situations and ensured job retention. ESS also shared instances where they intervened in situations where there were misunderstandings or unfair dismissal.

“Also talking to the boss and teaching the boss to understand why he [client] behaves in a way that he behaves and what kind of strategies will (be) needed for him to really be able to cope and go on to do the job there.” (ESS06/M).

The ESS also shared how they engaged potential employers and maintained **employer relations** to ensure healthy partnerships with employers. These strategies covered giving lunch-talks to establishments where clients could be placed in future. Expressing how she proactively engages employers was mentioned by, one ESS.

“Every few months I will ask the HR “do you want us to do a mental health talk?”... So I got one of my centre heads to go and present what’s schizophrenia, what’s depression, what’s medication and everything…There was about 10–12 of them who attended the lunch talk. So I think if we can focus on educating the bosses on what is really mental health, then I think there will be more opportunity.” (ESS15/F).

Throughout the interviews, ESS described how they viewed their role as one providing continual ‘**coaching and counselling**’ of their clients. The ESS often advised their clients outside their scope of work and responsibilities on a multitude of queries and concerns. These included providing informal suggestions on available employment resources for clients who did not wish to continue their engagement with formal support services or giving practical suggestions on their conduct at and outside of work.

“Because employment is the end goal you see, so you need to constantly motivate the client, which is where counselling comes in you know.” (ESS15/F).

The ESS also narrated roles and tasks that were not directly connected to employment placement. These ‘non-vocational’ tasks covered management of clients’ symptoms along with a multidisciplinary mental health professional team. Within this, narratives on how they ensured their clients wellbeing.

“We use those materials ..to talk about stress, how to identify stress. When you identify stress, what kind of emotions you have and how to really de-escalate... so like breathing exercise. We even teach things like mindfulness and what not. So these are all kinds of material stands that we use. How to manage their illness and also how to improve their so called life skills” (ESS06/M).

“We work very closely with other medical teams. We have multidisciplinary team meetings where each gives input on the person’s condition so we can make informed decisions about their health.” (ESS17/F).

Besides employers, clients and other healthcare professionals, ESS explained how it was necessary for them to **engage community partners**. These were services with social workers, counsellors, voluntary welfare organisations or law enforcement agencies active in the community. ESS often worked with such partners to improve access to job opportunities and support during employment, for example, through community-based services placed near clients’ workplaces to enable quick access and support for them.

“If they need some psychological support then they will be referred to a psychologist or community support that is more convenient.” (ESS19/F).

### Theme 2: Facilitators of Employment

Several experiences were shared by the ESS where their personal skills and disposition benefitted their clients or led to a successful employment experience. These attributes included **stewardship** which was perceived as being beneficial by the ESS. The ESS viewed themselves as most successful when they held a genuine belief in their clients’ capabilities, were patient and felt a responsibility to empower them through employment, as evident in the following narrative.

“So the patience, the understanding, and always leaving that door open (so) that they don’t feel that they are being judged, … then when they come back to you, it’s just “Ok, let’s restart the journey, what do you really want?” (ESS14/M).

However, the ESS also shared instances when they had to acknowledge employers’ motivations while hiring people with MHCs and the advantages of recommending the ‘right’ clients to them. At times this meant that some of their clients are not immediately placed in their preferred jobs, though in the long-run this fostered better relationships with employers and increased their willingness to continue hiring people with MHCs. This pragmatic understanding is best illustrated in the following quote:

“Now we have to balance with a real workplace productivity. Who [referring to employers] are there to run a business. So we have to take a lot into account, being comfortable with ambiguity. Greyness for because it’s not just about patients’ interest. But on a larger scheme we need to take care of other things as well…(possess) a degree of being flexible.” (ESS01/F).

A majority of the ESS expressed how **client-centeredness** was necessary. Learning about their clients as individuals and understanding their psychosocial context were deemed necessary to provide appropriate coaching and placement.

“Professionally trained as a counsellor, we do pay a lot of attention into you know, listening to what the client is struggling with and giving them the support and assistance, and helping them to see the hope.” (ESS18/F).

As expected, the ESS highlighted other factors that were beyond the control of the ESS. One such factor was their c**lients’ disposition** in terms of ideal traits such as being motivated or being open to suggestions were expressed as being beneficial from both personal and professional standpoints; these included being resourceful, relational, resilient and realistic. One participant attempted to provide an ideal description:

“They [clients] are motivated. They are open to suggestions or a bit more receptive to suggestions. I think two main traits. Yeah. So I think a lot of the cases are about motivation. It could be sudden, “I want to do this” then it dwindles very fast. So it needs to be like sustained and not like overly motivated at one point of time that kind of thing. Yeah. Then, they are receptive to suggestions and they would seek help, or seek assistance.” (ESS20/F).

While expressing their thoughts on the importance of clients having a right attitude towards work, several ESS shared their thoughts on having sustained motivation and preparedness to work, being willing to receive employment support, and putting concerted personal effort to attain employment, as well as the importance of a positive attitude to achieve employment success, as seen below.

“She will do her homework. She will try it out. If it doesn’t work, she will tell you it doesn’t work. Then we will just try something new. For her, (she had a) learning attitude, so it was very good.” (ESS20/F).

**Clients’ perception of their illness** was also expressed to be important as it allowed them to set realistic and sustainable employment goals. ESS also raised an interesting perspective of how it was important for the clients’ to not regard their illness as a disability and those who managed to look past their illness were perceived to be successful in their attempts at employment.

As expressed by the ESS,

“Readiness was there to accept (his condition) and now he’s in a workplace quite long. Because he is working on those (symptomatic) issues. “(ESS09/F).

“I find that those who don’t harp on their illness... they can do better.” (ESS07/F).

Clients’ s**upport networks** were consistently expressed as vital to their employment success, particularly as a means for supportive communication and emotional strength which helped them in retaining their jobs. These related to clients’ family, relatives, close friends as well as colleagues.

The ESS also shared how other stakeholders such as governmental organizations, community partners and social service agencies played key roles in supporting employment of people with MHCs- through policies on fair practices, helpful schemes such as subsidy for commuting to workplace and social assistance for the people with MHCs despite not always being in direct contact with them. Lastly, the ESS also recounted the use of technology and social media or networking platforms such as WhatsApp groups that enabled better access to employers and knowledge on job openings for their clients.

“They do better when (they) find support through the peers.” (ESS17/F).

### Theme 3: Challenges

During the interviews, the ESS expressed several challenges that were within and outside their personal boundaries. Expressing their **personal challenges**, the ESS narrated their struggles while **balancing expectations of clients and employers**. ESS often encountered clients who preferred to acquire administrative jobs or those with flexible working hours. On one hand, ESS expressed that they had to constantly persuade or balance the expectations of their clients and place them in jobs, while on the other; they were mindful of the employers’ demands and thereby struggled to create a good impression. These are best reflected in the following narrative.

“I think it’s good to know what does the patient really want, and the ability to match their wants, their needs to the market and also to their ability. So the major challenge I think for anyone of us here is that you may think you know that this person is not suitable for this job, but this person insists that I want this job. So how to balance that and everything is the major challenge” (ESS14/M).

A number of ESS faced professional **identity struggles** in their course of work. As narrated by one ESS,

“I am not very keen to use the word employment specialist because the employment specialist designation suggests things that are very related to work. I struggled with this title for a long time, it was like 4 years (ago) and now I moved on to something else, but I am still same in that sense … The minute you call yourself an employment specialist, two things happen. Our self, the staff members say ‘oh this is not my job, my job is just to provide work’. But you cannot just deal with their work, there’s other issues!” (ESS18/F).

Some of them attributed it the multiple tasks they had to undertake every day, that often blurred the lines between their primary job description and commitment towards their clients. Together with professional identify challenges, ESS we spoke to in our study encountered a **dilemma of disclosure of clients’ mental condition** to potential employers. A number of ESS, particular those who were not directly affiliated with mental healthcare institutions or who were engaging employers via other external placement agencies, brought up the dilemma they faced with regards to advising clients on disclosing their condition to employers. Generally, clients’ attitude and opinion on disclosure determined how ESS approached this issue.

“The moment you declare that you have a MHC, you (have) potentially narrowed your chance of clinching the job” (ESS05/M).

“(It) depends on what the client wants. Whether they want to disclose that they have this ..um.. mental health issue or whether they choose not to disclose” (ESS16/F).

Not disclosing their clients’ diagnoses to employers, however, seemed to pose a dilemma and challenge to the ESS in terms of assisting the persons with MHCs secure employment as evident in this narrative.

“If they don’t want to tell the employers, then of course we’ll limit them from direct placement. But then, they cannot tap onto our current employer database” (ESS04/F).

 A handful of ESS mentioned how their clients had benefited through disclosure as they were hired through proper (via mental health employment specialist or placement services) channels and processes, resulting in a supportive workplace environment.

“If you disclose, and it happens to be a company that’s pro-MHC, they do have the necessary support. They could (help)… Life would be easier for you when you work there. Right? The support will be there, the bosses, the supervisors will keep a lookout for you, even help you to manage your stress level” (ESS08/F).

Beyond their personal limitations and struggles, ESS expressed difficulties pertaining to their clients, employers and the socioeconomic environments. **Managing job expectations of clients** was expressed as a routine challenge for the ESS because in addition to being unwell, a number of their young clients were also unsure of their work capabilities and often lacked the requisite experience, thus making it more difficult for ESS to match jobs for them. Their clients also often seemed to rely on the opinions of others, such as their parents, to negotiate with ESS and the entire process at times resulted in frustration when their expectations are not met. For example, one ESS said,

“They will say that my parents said this is too low, too low pay, or this type of job is not suitable for me.” (ESS03/M).

These challenges were further compounded when the younger clients lacked pre-existing job experience or were experiencing medication side effects or had severe symptoms, particularly with respect to sustaining employment.

In addition, the ESS narrated how **other factors** that affected employment prospects of their clients posed challenges for them while performing their duties. ESS felt that although young persons with MHCs generally preferred finding jobs from the open market, they had limited options due to perceived stigma and high expectations of employers. Another challenge for the ESS was to ensure their clients were treated fairly at work. The ESS shared instances where they suspected that their clients were underpaid or bullied at the workplace.

“They are already underpaid so it’s a free labour for them…I think my experience is the client is not treated with respect…they will shout at them, I remember (when) I went there he was shouting at the clients.” (ESS13/M).

A system-wide challenge expressed by the ESS was the lack of adequate job opportunities for their clients which they attributed to limited listings in the jobs databases they accessed. This was expressed more strongly in relation to positions such as unskilled or manual work, or entry level opportunities for clients with lower academic qualifications.

While describing events and experiences from their routine work, it was evident that ESS felt organizations had a mind-set of maximizing productivity at work, due to which employers were not keen to hire people with MHCs, in general. Participants expressed that although some employers are open to hiring their clients, they may either not be prepared to reduce work efficiency or were wary due to their lack of knowledge and resources to manage people with MHCs.

“There are employers that are quite compassionate. So they are willing to hire them but they [employers] don’t really know how to adapt the role to suit them [clients] better. So like, they [employers] have the heart but then something in between didn’t appear [Referring to appropriate processes/ways to support employees with MHCs], so they don’t really know how to adapt. So (it becomes a) wrong fit in terms of job. Then that’s like a bit of a pity.” (ESS20/F).

While describing their challenges, many ESS mentioned ways to counter these and improve employment opportunities for people with MHCs. Suggestions included ways to expand the pool of employers, enhance employment support services and community engagement and implementing societal level initiatives to de-stigmatize mental conditions. Verbatim accounts of these suggestions are listed in Table 3.

**Table 3:** Employment Support Specialists’ feedback and suggestions to improve employment opportunities for people with mental health conditions

Categories	Sample suggestions and quotes
Increase the pool of employers	Reaching out to big organisations or the government for jobs. “Outreach for employers... I don’t know, [National Centre] personnel who can build relationship with organisations. Big organisations… They govern a lot of things.”—ESS01/F
	National Centre to lead employment support for people with MHCs “[National Centre] could also start to employ not so much on employment specialist, but I’m just saying that, our clients to do some simple jobs.”—ESS03/M
	Develop a pool of employers who are truly willing to employ people with MHCs “Pool of employers who are understanding and willing to go the extra mile to support person with mental health challenges. I think we need to really (have) you know, (such) a pool.”—ESS06/M
	Provide more financial incentive for employers to hire “Tap on the government’s schemes where a company can employ these people as interns, and then the government pay(s). It’s a policy for everybody who has no job.”—ESS14/M
Employment support for people with MHCs	Need for a safe working environment for people with MHCs “Work is therapeutic, yes. But it has to be supervised and it has to be a safe environment.”—ESS10/M
	Provide free of charge employment support services “[Voluntary Welfare Organisations] normally initially (used to) say charge $10, I said no it’s not going to work. So when we stopped charge [referring to charging fees], and it’s [ESS service] free…more (would) come.”—ESS13/M
	Improve socialization process at the workplace “I think if the socialization process can be improved for them, they will be much more (at ease at work). Their recovery journey will be much better.”—ESS14/M
	Provide supported employment “Supported employment may also be very helpful for the lower functioning clients, they provide support. So then they [ESS] go down to the employer and then uh, they look at how their client is doing and to provide support for the clients.”—ESS19/F
Engagement of formal and informal community service providers	Refer clients to community partners “Whether or not can we refer some of the easier cases to the community partners? Because a lot of our clients need the [employment support] service but they are not willing to associate with job club because they don’t want to be linked to [National Centre], they don’t want the stigma to be with them.”–ESS02/F
	Integrated support “We have to think about rehab as a department and program in [National Centre] and to see how we can link the inpatient, the outpatient clinic and the community all together and how do we interact with each other.”—ESS11/M
	Develop community support “We always think (that) the government’s got to be the one (to help), but there are a lot of people out there (who are) able to provide the means to (employment). Maybe just set up an institution or company where they can actually allow real training, you know like from the cleaner to the admin to the, you know to anything. It could be just like a bookshop or it could be a small little café, you know people who are willing to fund and put this people where we can actually really train (them).”—ESS18/F
Training	More training for people with MHC to understand the current job market “These are somethings.. (such as) could be a formal training, but yet can be also (be used) for some experience”—ESS08/F
	More training for ESS to improve skills “It’ll be good to go for further training for ourselves as well for some of the things (that) we may (have) forgotten or we just need that fresh breath of uh whatever new training, techniques that people have used and have been helpful to them to, to actually share (these) with us.”—ESS16/F
Societal level initiatives	Address stigma of mental illnesses “For a start, I think there’s a lot of work that needs to be done in the community itself. Yeah, I think employers need to be educated about this mental illness. It’s not as bad as what they may think ..(it) is violent,. So, if there’s some sharing (with the employers) we should be able to get more help.”—ESS03/M
	Advocacy “My observation with autism, somehow it moved very fast but because they got a champion… Can we find a champion? I mean since that has worked, seems that with [Celebrity’s name]… Is there someone or is there a model that works for mental illness or disability?”—ESS17/F
	Address the issue around disclosure at the time of employment “If the ministry can really dispense off the need for people to declare”—ESS18/F

*MHCs* Mental health conditions, *ESS* employment support specialists, *F* female participant, *M* male participant

## Discussion

Through the narratives of the ESS, the study explored tasks they routinely undertake in the course of their work in Singapore and the various facilitators and barriers experienced by them while supporting young people with MHCs secure employment. Overall, the findings show that the ESS held multiple roles throughout the course of their work. In performing these roles, the ESS adopted supported employment and IPS models that are used in North America, Australia, and Europe, whereby their roles are streamlined and catered to the needs of their clients [14, 15]. Our participants mentioned that they provided counselling and advice regularly to their clients. This covered benefits’ counseling whereby they ensured fair employment and other benefit planning to assist their clients in job selection decisions, in line with the IPS approaches [15]. However, some also provided counselling on the appropriate time to take up employment or even asked their clients to defer placements for health reasons. This varies from the IPS principle of rapid job placement and might be influenced by the Asian cultural setting where clients seek and expect additional guidance from care providers and professionals. In addition, the ESS integrated with mental health institutes were engaged more closely in the clinical care planning and follow-up of their clients. This was similarly observed in other developed countries such as the USA [16], where integrated care models are a norm. While the placement of employment specialists in clinical teams can offer opportunities for more holistic support to clients, it can also impose additional workload, a lack of clarity for the ESS regarding their professional roles and present dissimilarities in non-healthcare-based employment support services and community-based support services. Further in-depth understanding of the ESS roles that are not directly related to employment is necessary to avoid role duplication and strain.

The ESS leveraged on personal- and client-related strengths and attributes to ensure successful placements of their clients. For example, ESS believed stewardship and client-centeredness were beneficial to the vocational success of their clients that also resulted in not only better collaborations within their teams, but also with clients and potential employers. This perspective is supported by results from studies among American, Canadian and Dutch ESS that found that a client-centered approach and better relationships between the ESS and employers or supervisors resulted in successful job placement of people with MHCs [12, 33]. Our exploratory study indicates that such approaches and skillset among the ESS could offer greater potential to effective service provision and form synergistic communications and relationships with clients and employers. Emphasizing this approach to ESS may thus improve their effectiveness in the field.

Another aspect believed to be beneficial to clients’ job placements was the relationship between the ESS and other community or mental health professionals and services. Successful collaboration between ESS and the mental health providers forms the foundation for effective IPS and emphasizes having an ‘integrative’ over a ‘collaborative’ model of support. This is perhaps a reflection of the local system prevalent in the relatively small nation, that emphasizes collaboration across various sectors in order to avoid duplication and maximize benefits to the population. About two thirds of the participants in this study were co-located at a tertiary psychiatric hospital. Liaising with clinical teams formed a key non-vocational role of the ESS in this study, which at times posed a challenge in terms of managing clients’ expectations in line with clinical decisions. Likewise, challenges in building effective partnerships between different agencies and ESS were the basis of a study in USA [16]. To reduce conflicts between the ESS and mental health teams, the authors recommended involving ESS in mental health treatment team meetings and highlighted the need to periodically assess the quality of collaboration between ESS and clinical teams, which should be considered for enhancing processes within ESS teams in Singapore.

However, our study also highlights several challenges faced by the ESS. In this study, the ESS mentioned placing more efforts for clients with specific job preferences and those with no prior work experience. Moreover, within this group, those with low motivation or unclear expectations were found to be particularly challenging to place. Results indicate that a strengths-based approach to job placements and appropriate training for the ESS should be incorporated in the current processes to enable ESS to cater to this clientele. ESS also mentioned that many employers were not receptive to hiring people with MHCs; reasons often cited were stigma associated with MHCs, productivity concerns, and inadequate or lack of support for employees experiencing psychiatric symptoms. This further limited the options for clients’ job placements. ESS were therefore left with limited resources and pool of employers. Since the initiations of this study, several ministerial levels efforts have been implemented in Singapore to improve employment opportunities for MHCs. This study indicated the extent of the problems that may need concerted efforts in the local setting. To improve service provision ability of the ESS, it is also necessary to actively seek new employers or organizations and to provide counselling or other support to empower them in managing symptoms.

An underlying limitation often expressed by the ESS in the current study was the dilemma of disclosure of clients’ MHC to employers. In the context of Singapore, until the more recent enforcement of TAFEP guidelines and other enhanced de-stigmatization efforts by the government, disclosure of job seekers’ health conditions including history of any psychiatric conditions to their potential employers had been an area of debate; and some uncertainly still remains on this issue [27]. By virtue of the placement of the ESS who were based in a tertiary psychiatric institution, it is implicit that diagnoses of clients with supported employment services are disclosed to employers. However, some ESS may be providing support that is limited to vocational training and job preparation or utilizing other private employment agencies; thus, having a choice not to disclose their diagnosis. This could explain the narratives of the ESS in our study. Disclosure of illness was as expected perceived to be “double-edged sword” by the ESS in our study. Similar findings were reported in a recent study conducted among people with MHCs in Netherlands where human resource and work reintegration professionals identified discrimination and stigma resulting from disclosure as a key disadvantage and deterrent to disclosure [34]. On the other hand, advantages included ‘improved relationships, authenticity, work environment support, and friendly culture’ experienced by their clients. The authors also highlighted the process of disclosure being influenced by other factors such as considerations on to whom, how and what to disclose. Taken in conjunction with the present study, these reports further elucidate the inherent dilemma of disclosure, indicating a need to address this regular experience of the ESS.

According to the participants of this study, potential strategies to improve employment prospects for people with MHCs include having effective anti-stigma campaigns, greater engagement of the job sector and employers, referrals of clients to community partners to address issues around disclosure, and enhanced training and skills development of the ESS. ESS trained in cognitive remediation have been found to be more effective in helping clients who had otherwise not benefited from routine supported employment [35], indicating one possible direction for training of ESS in Singapore. In relation to training and effective employment support in the current setting, innovative approaches such as use of technology, and mobile computer-based training and engagement interventions have demonstrated potential. Examples include a computerized intervention for improving contact between clients and ESS [36], and the use of virtual reality to facilitate job interview skills training [37]. It may be worthwhile to explore some of these approaches to improve ESS services. Another gap that was evident in this study was the need to enable employers and organizations to hire more persons with MHCs. The interviewees narrated how employers were ill equipped to adapt jobs for their clients despite clients having considerable support from their ESS. In this regard, appropriate employer training could enable ESS in this role function. Alternatively, incorporating trained occupational or vocational therapists in the job modification processes could be beneficial.

As policy makers seek to understand and improve processes for people with MHCs in their transition to employment, community-wide interventions [38] and hospital-based employment initiatives such as the Supported Employment (SE) programme [39], IPS [15, 40], family-aided assertive community treatment [41] and integrated case management including peer support specialist teams [42], are being implemented and tested in various countries. Systemic challenges to optimal employment support for people with MHCs identified consistently across studies include policy environments that may limit fund availability to community-based organizations and lack effective integration of efforts from community and clinical services [43], and higher cost of service delivery [40]. In the current study, participants shared variability in their frequency and duration of client engagement. This observation lends to common limitations of job rehabilitation models, i.e. higher cost and resource burden associated with ‘unlimited’ provision of such services [40]. To counter this, a model of ‘placement budgets’ for supported employment that set predefined maximum time budget for the duration from job seeking to competitive employment was tested in Switzerland [44] and found that 40 hours of support was effective in securing jobs for over half of the clients within the first 12 months of support. In contrast to this, a study that examined ‘follow-along support’ on job duration found the frequency of ESS contact was positively related with job retention [45], presenting an uncertainty on this issue. The current study provided areas that could be proritised for adoption of new models and processes in order to aid ESS in Singapore.

The results of this study, however, need to be considered in light of some limitations. Firstly, the study focused on the perspectives of ESS regarding employment support to people with MHCs, purposefully limiting the scope to employment of younger clients. Although interviewers were mindful of reminding and probing for perspectives related to clients in this age group, it is possible that some of the experiences extend beyond and relate to older clients. Secondly, this study was based on ESS’ perceptions and not observed practices, hence it is possible that more notable experiences were recounted during the interviews than those frequently experienced in routine practice. Social desirability bias may have affected some responses wherein some unpleasant experiences with clients or employers, or system level challenges were under reported, particularly among ESS working in the tertiary psychiatric organization where the research was carried out. For example, only a few participants mentioned serious conflicts with employers or clients. In addition, the majority of participants in this study were working at a tertiary psychiatric institution and catered to more clients with MHCs such as psychosis or schizophrenia. It is therefore possible that their narratives referred more to clients with higher demands, and they might not have expressed experiences of other routine interactions during our interviews given that we aimed to understand their challenges. However, we believe that the inclusion range of participants from other organizations helped address this limitation and allowed representation of general experiences of ESS in course of their work. Our research was also not designed to identify variations across professionals or organizational factors, which warrant follow-up and targeted studies in these groups in future. For example, we observed that the older ESS, particularly those engaged in non-healthcare organizations, expressed more limitations. In addition, although we made great efforts to include interviewes that worked with more severely affected clients, our sampling strategy based on self-referral could have led to under-reporting of experiences relevant to more marginal and unwell clients under other health sectors. Lastly, the terms ‘employment’, ‘employers’ and ‘MHCs’ were used broadly in the study and no attempts were made to factor in their specific types or categories. It is, therefore, possible that ESS experiences reflected in this study draw attention to wider overarching issues while missing more targeted information. These limitations notwithstanding, an important strength of the study is that participants belonged to multiple organizations and occupational backgrounds, improving transferability of these results to other settings. Furthermore, the study achieved adequate data saturation, thereby improving confidence in its trustworthiness.

In summary, this study identified various personal and external barriers faced by the ESS that can be addressed to optimize and improve effectiveness of employment support for people with MHCs. Successful implementation of employment support strategies requires a better understanding of the process, its challenges and potential areas for improvement from various perspectives. This study presents perspectives of ESS and provides a starting point for deeper investigation into their work in Singapore and elsewhere.

## Electronic supplementary material

Below is the link to the electronic supplementary material.

Electronic supplementary material 1 (DOCX 16 kb)

## References

[1] Charette-Dussault É, Corbière M (2019). An integrative review of the barriers to job acquisition for people with severe mental illnesses. J Nerv Mental Dis.

[2] Chong SA, Verma S, Vaingankar JA, Chan YH, Wong LY, Heng BH (2007). Perception of the public towards the mentally ill in developed Asian country. Soc Psychiatry Psychiatr Epidemiol.

[3] Subramaniam M, Abdin E, Picco L, Pang S, Shafie S, Vaingankar JA, Kwok KW, Verma K, Chong SA (2017). Stigma towards people with mental disorders and its components: a perspective from multi-ethnic Singapore. Epidemiol Psychiatr Sci.

[4] Liljeholm U, Bejerholm U (2019). Work identity development in young adults with mental health problems. Scand J Occup Therapy.

[5] Filia K, Jackson H, Cotton S, Killackey E (2019). Understanding what it means to be socially included for people with a lived experience of mental illness. Int J Soc Psychiatry.

[6] Arns PG, Linney JA (1993). Work self and life satisfaction for persons with severe and persistent mental disorders. Psychosocial Rehabil J.

[7] Cook JA, Razzano L (2000). Vocational rehabilitation for persons with schizophrenia: recent research and implications for practice. Schizophr Bull.

[8] Cook JA, Blyler CR, Leff HS, McFarlane WR, Goldberg RW, Gold PB, Mueser KT, Shafer MS, Onken SJ, Donegan K, Carey MA, Kaufmann C, Razzano LA (2008). The employment intervention demonstration program: major findings and policy implications. Psychiatr Rehabil J.

[9] Schneider J, Boyce M, Johnson R, Secker J, Slade J, Grove B, Mike F (2009). Impact of supported employment on service costs and income of people with mental health needs. J Mental Health.

[10] Cobigo V, Stuart H (2010). Social inclusion and mental health. Curr Opin Psychiatry.

[11] Bond GR, Becker DR, Drake RE, Rapp CA, Meisler N, Lehman AF, Bell MD, Blyler CR (2001). Implementing supported employment as an evidence-based practice. Psychiatric Serv.

[12] Corbière M, Zaniboni S, Lecomte T, Bond G, Gilles PY, Lesage A, Goldner E (2011). Job acquisition for people with severe mental illness enrolled in supported employment programs: a theoretically grounded empirical study. J Occup Rehabil.

[13] Lo TL, Warden M, He Y, Si T, Kalyanasundaram S, Thirunavukarasu M, Amir N, Hatim A, Bautista T, Lee C, Emsley R, Olivares J, Yang YK, Kongsakon R, Castle D (2016). Recommendations for the optimal care of patients with recent-onset psychosis in the Asia-Pacific region. Asia-Pacific Psychiatry.

[14] Mueser KT, McGurk SR (2014). Supported employment for persons with serious mental illness: current status and future directions. L’Encephale.

[15] Becker DR, Drake RE (1993). A working life: the individual placement and support (IPS) program.

[16] Swanson SJ, Courtney CT, Meyer RH, Reeder SA (2014). Strategies for integrated employment and mental health services. Psychiatr Rehabil J.

[17] Bond GR, Drake RE, Becker DR, Mueser KT (1999). Effectiveness of psychiatric rehabilitation approaches for employment of people with severe mental illness. J Disabil Policy Stud.

[18] Oshima I, Sono T, Bond GR, Nishio M, Ito J (2014). A randomized controlled trial of individual placement and support in Japan. Psychiatr Rehabil J.

[19] Tsang HWH, Fung KM, Leung AY, Li SM, Cheung WM (2010). Three year follow-up study of an integrated supported employment for individuals with severe mental illness. Aust N Zeal Psychiatry.

[20] Wong KK, Chiu LP, Tang SW, Kan HK, Kong CL, Chu HW, Chiu SN (2004). A supported employment program for people with mental illness in Hong Kong. Am J Psychiat Rehabil.

[21] Drake R, Bond G (2008). The future of supported employment for people with severe mental illness. Psychiatr Rehabil J.

[22] Lee HL, Hwang EJ, Wu SL, Tu WM, Wang MH, Chan F (2018). Employment outcomes after vocational training for people with chronic psychiatric disorders: a multicenter study. Am J Occup Ther.

[23] Labour E. Wages and Productivity. Ministry of Manpower (MOM), Singapore statistics. https://www.singstat.gov.sg/find-data/search-by-theme/economy/labour-employment-wages-and-productivity/latest-data. Accessed 31 Dec 2019.

[24] How MOM. deals with employment discrimination. A report by the Ministry of Manpower (MOM), Singapore. https://www.mom.gov.sg/-/media/mom/documents/parliament/how-mom-deals-with-employment-discrimination-final.pdf. Accessed 31 Dec 2019.

[25] National Council of Social Service. Singapore website. https://www.ncss.gov.sg/. GatewayPages/Social-Services/Persons-with-Mental-Health-Issues/Employment. Accessed 31 Dec 2019.

[26] Presidents challenge 2019 launched with focus on mental health. The Straits Times, News article dated 30 January 2019. https://www.straitstimes.com/singapore/presidents-challenge-2019-launched-with-focus-on-mental-health. Accessed 31 Dec 2019.

[27] Advocacy group urges firms to eliminate or reword declaration; seeks help from Tafep. The Straits Times, News article dated 27 Sep 2016. https://www.straitstimes.com/singapore/call-to-remove-mental-health-query-on-job-forms. Accessed 31 Dec 2019.

[28] Tan BL, Li ZY, Tan CHM (2016). Evaluation of a national supported employment programme for people with psychiatric conditions. Br J Occup Therapy.

[29] Braun V, Clarke V (2006). Using thematic analysis in psychology. Qual Res Psychol.

[30] Nvivo software. https://www.qsrinternational.com/nvivo-qualitative-data-analysis-software/about/nvivo. Accessed 17 Oct 2020.

[31] MacQueen KM, McLellan E, Kay K, Milstein B (1998). Codebook development for team-based qualitative analysis. Cult Anthropol Methods.

[32] Green J, Thorogood N. Qualitative methods for health research. Sage; 2018.

[33] Taylor AC, Bond GR (2014). Employment specialist competencies as predictors of employment outcomes. Commun Ment Health J.

[34] Brouwers EPM, Joosen MCW, van Zelst C, Van Weeghel J (2020). To disclose or not to disclose: a multi-stakeholder focus group study on mental health issues in the work environment. J Occup Rehabil.

[35] Teixeira C, Mueser KT, Rogers ES, McGurk SR (2018). Job endings and work trajectories of persons receiving supported employment and cognitive remediation. Psychiatric Serv.

[36] Haslett WR, McHugo GJ, Bond GR, Drake RE (2014). Use of software for tablet computers to promote engagement with supported employment: results from an RCT. Psychiatric Serv.

[37] Bell MD, Weinstein A (2011). Simulated job interview skill training for people with psychiatric disability: feasibility and tolerability of virtual reality training. Schizophr Bull.

[38] Seebohm P, Secker J (2003). Increasing the vocational focus of the community mental health team. J Interprof Care.

[39] Sherring J, Robson E, Morris A, Frost B, Tirupati S (2010). A working reality: evaluating enhanced intersectoral links in supported employment for people with psychiatric disabilities. Aust Occup Ther J.

[40] Dixon L, Hoch JS, Clark R, Bebout R, Drake R, McHugo G, Becker D (2002). Cost-effectiveness of two vocational rehabilitation programs for persons with severe mental illness. Psychiatric Serv.

[41] McFarlane WR, Dushay RA, Deakins SM, Stastny P, Lukens EP, Toran J, Link B (2000). Employment outcomes in family-aided assertive community treatment. Am J Orthopsychiatry.

[42] Mowbray CT, Rusilowski-Clover G, Arnold J, Allen C, Harris S, McCrohan N, Greenfield A (1994). Project WINS: integrating vocational services on mental health case management teams. Community Ment Health J.

[43] Buizza C, Pioli R, Lecchi S, Bonetto C, Bartoli A, Taglietti R, Ghilardi A, Riva E (2014). Mental disorders and work integration: a retrospective study in a northern Italian town. Clin Pract Epidemiol Ment Health.

[44] Rössler W, Kawohl W, Nordt C, Haker H, Rüsch N, Hengartner MP (2020). ’Placement budgets’ for supported employment: impact on employment rates in a multicentre randomised controlled trial. Br J Psychiatry.

[45] Bond GR, Kukla M (2011). Impact of follow-along support on job tenure in the individual placement and support model. J Nerv Ment Dis.

