# Supplementary material for: Roles, Facilitators and Challenges of Employment Support Specialists Assisting Young People with Mental Health Conditions
Source: J Occup Rehabil. 2020 Oct 22;31(2):405–18. doi: 10.1007/s10926-020-09930-x (PMC8172398; doi:10.1007/s10926-020-09930-x)
Supplement: Supplementary file 1 — Electronic supplementary material 1 (DOCX 16 kb) [file 10926_2020_9930_MOESM1_ESM.docx]

**Supplementary file 1**

**Interview guide**

Aim: To understand employment support specialists’ experiences and challenges of providing service to young clients with mental health conditions, and their understanding of clients’ employment-related needs.

***General history:***

- Can you tell me about yourself and about the job you do at your organisation?
- Can you tell me more about your job of assisting young persons with mental health conditions with employment? Can you briefly describe their needs?

***Information regarding their role in employment:***

- Please share your experience of doing this job.
- How do you identify suitable jobs for them?
- What role do you play once the person has taken up a job?

If not mentioned in prior answer, have you ever had to deal with a person who lost his job due to issues related to his mental health issues – how did you deal with it?

- Based on your own experience what skills must a good employment support specialist possess? How do you think such skills can be acquired?
- Overall how would you describe the nature of your interactions with young persons with mental health conditions

***Thoughts on employing young people with mental health conditions:***

- What are your thoughts regarding assisting young persons with mental health conditions with employment?
- What do you personally think are some of the challenges associated with employing someone with a mental health condition?
- Can you tell us about any positive and/or negative experiences associated with assisting young persons with mental health conditions with employment?
